# Comparative analysis of CDR3 regions in paired human αβ CD8 T cells

**DOI:** 10.1002/2211-5463.12690

**Published:** 2019-07-12

**Authors:** Kun Yu, Ji Shi, Dan Lu, Qiong Yang

**Affiliations:** ^1^ Department of Breast and Thyroid Surgery Zhejiang Provincial People's Hospital People's Hospital of Hangzhou Medical College Hangzhou China; ^2^ Department of Breast and Thyroid Surgery TongDe Hospital of Zhejiang Province Hangzhou China; ^3^ Department of Rehabilitation TongDe Hospital of Zhejiang Province Hangzhou China

**Keywords:** CD8 T cell, CDR3 region, T‐cell receptor, TCR pairing

## Abstract

The majority of human CD8 cytotoxic T lymphocytes express αβ T‐cell receptors that recognize peptide–MHC class I complexes. Considerable attention has been devoted to TCR β repertoires, but study of TCR α chains has been limited. To gain a better understanding of the features of CDR3α and CDR3β in paired samples, we comprehensively analyzed 776 unique paired αβ TCR CDR3 regions in this study. We found that (I) the CDR3 length among paired αβ TCRs had a fairly narrow distribution due to random assortment of CDR3 length in alpha and beta chains; (II) nucleotide deletions among CDR3 regions were positively correlated with insertions in both α and β TCRs; (III) the CDR3 loops of both α and β chains contained an abundance of charged/polar residues and the CDR3 base regions contained a conserved motif; and (IV) the occurrence of Gly was CDR3 length‐ and position‐dependent in both chains, whereas the frequency of Ser at positions 106 and 107 was positively correlated with CDR3 length in TCR β. Overall, the amino acids in CDR3 loop regions were significantly different between TCR α and β, which suggests a distinct role for each chain in the recognition of antigen–MHC complexes. Here, we have provided detailed information on CDR3 in paired TCRs expressed on human CD8+ T cells and established the basis of a reference set for αβ TCR repertoires in healthy humans.

**Abbreviations**
CDR3: complementarity‐determining region
CMV: cytomegalovirus
HLA: human leukocyte antigen
MHC: major histocompatibility complex
RACE: rapid amplification of cDNA end
TCR: T‐cell receptor

Antigen‐specific CD8+ T cell‐mediated immune responses depend on the appropriate recognition of the αβ T‐cell receptor (TCR) against peptide–major histocompatibility class I (MHC I) molecule complexes 1, 2, 3. The binding site of the TCR includes three complementarity‐determining regions (CDRs) for each chain in which CDR3 is the most diverse and important CDR in antigen recognition. The CDR3 regions from TCR α and β chains are in contact each other and form the center of the antigen‐binding site. Conformational flexibility of the CDR3 regions as well as the composition of amino acids play an important role in determining the antigen specificity and binding affinity of the TCRs, including the recognition of different peptide–HLA ligands 4, 5. Several small datasets have shown that the utilization of amino acids within the CDR3 region is nonrandom. For example, charged or polar residues were found to be prevalent in α chains, and glycine was frequently observed in β chains irrespective of high CDR3 diversity 6, 7. In addition, the results of alanine scanning mutagenesis studies indicated that a single substitution within the CDR3 region can play a major role in conformational and/or functional changes of TCRs 8, 9. Taken together, these observations emphasize the importance of the distribution of amino acid residues on the effect of TCR binding stability and/or flexibility within the CDR3 regions. However, the observations mentioned above were based on very small‐scale datasets and a few different studies, so a comprehensive analysis with large datasets was needed to further investigate the features of the CDR3 region, and we believe the general features of the CDR3 region can provide us with better baseline knowledge for TCR engineering.

Human leukocyte antigens (HLAs) play an important role in T‐cell receptor positive and negative selection 10, 11. In the current analysis, we collected a large single‐cell TCR dataset from a single published work by Sun *et al*. 12, which includes the largest dataset to date from an Asia population group. Because Japanese and Chinese populations have very similar HLA distributions 13, we selected this dataset for further analysis because it could provide more useful information on the Chinese population. We investigated paired CDR3 length distribution, nucleotide insertions/deletions, correlations with germline sequences in the CDR3 region, amino acid usage in the CDR3 loop regions, and the potential for intrinsic pairing of αβ TCRs. We expected to obtain the fundamental properties for TCR repertoire paring in CD8 T cells, which could then be utilized as a base for assessing the TCR repertoire in patients with infectious diseases or tumors as well as improving TCR engineering for adoptive immunotherapy.

## Materials and methods

### Sample datasets

The dataset was publicly available on GenBank, and the accession numbers for the sequences examined in this study are AB976719 to AB977494 for the alpha chain (776 samples) and AB977495 to AB978270 for the beta chain (776 samples). The sequence IDs are in column B and column AA of Table S1 for alpha and beta chains, respectively. From the study they described in their supplemental information and GenBank data, we confirmed that there are more than 1.5K sequences have been reported. We verified that all 776 samples represent unique pairings and all expanded T‐cell clones were removed from current analysis. All sequences are in frame. The donor information was described in a previous study and through personal contact with authors 12. In addition, the TCR alpha and beta chains from previous studies were amplified from mRNA using 5′ rapid amplification of cDNA end (RACE) and multiplex PCR methods from single CD8 T cells and sequenced by the standard Sanger sequencing method 12.

### TCR sequence analysis

All the TCR sequences were extracted from GenBank and analyzed with the IMGT/V‐QUEST tool (http://www.imgt.org/IMGT_vquest), and all the parameters, including the CDR3 definition, CDR3 length, and nucleotide addition and insertion, as well as the properties of the amino acids, followed the nomenclature in the IMGT database 14. The sum of CDR3 length is calculated from the sum of CDR3 length from alpha chain and the corresponding CDR3 length from paired beta chain.

### Amino acid composition and Shannon entropy

The amino acid composition at each position was generated with weblogo 3.4 (http://weblogo.threeplusone.com/create.cgi), and the color of each amino acid was determined according to the chemical properties, including polar (G, S, T, Y, C), neutral (Q, N), basic (K, R, H), acidic (D, E), and hydrophobic (A, V, L, I, P, W, F, M) properties, which were displayed as green, purple, blue, red, and black, respectively. The variability of each position was calculated by Shannon entropy according to a previously described method 15. The detailed amino acid composition of alpha chain and beta chain is shown in Tables S2 and S3.

### Biochemical property calculation

All the physicochemical values and amino acid properties were obtained from the IMGT database (http://www.imgt.org/IMGTeducation/Aide-memoire/_UK/aminoacids/IMGTclasses.html) and were described previously 14. In this analysis, the first three and the last amino acid were removed from the CDR3 region as described previously 16. The 20 amino acids were classified according to the IMGT database. There are 3 ‘Hydropathy’ classes: hydrophobic (A, C, I, L, M, F, W, and V), hydrophilic (R, N, D, Q, E, and K), and neutral (G, H, P, S, T, and Y). The hydropathy value of each amino acid was given by the Kyte–Doolittle score from IMGT. In addition, there are 2 IMGT ‘Polarity’ classes, polar (R, N, D, Q, E, H, K, S, T, Y) and nonpolar (A, C, G, I, L, M, F, P, W, V), as well as 3 IMGT ‘charge’ classes that included positive charge (R, H, K), negative charge (D, E), and uncharged (A, N, C, Q, G, I, L, M, F, P, S, T, W, Y, V). To systematically normalize each value, the sum of each value was divided by CDR3 length. The detailed calculation of each CDR3 region is shown in Table S4.

### Graph illustrator

**Figure 1:**
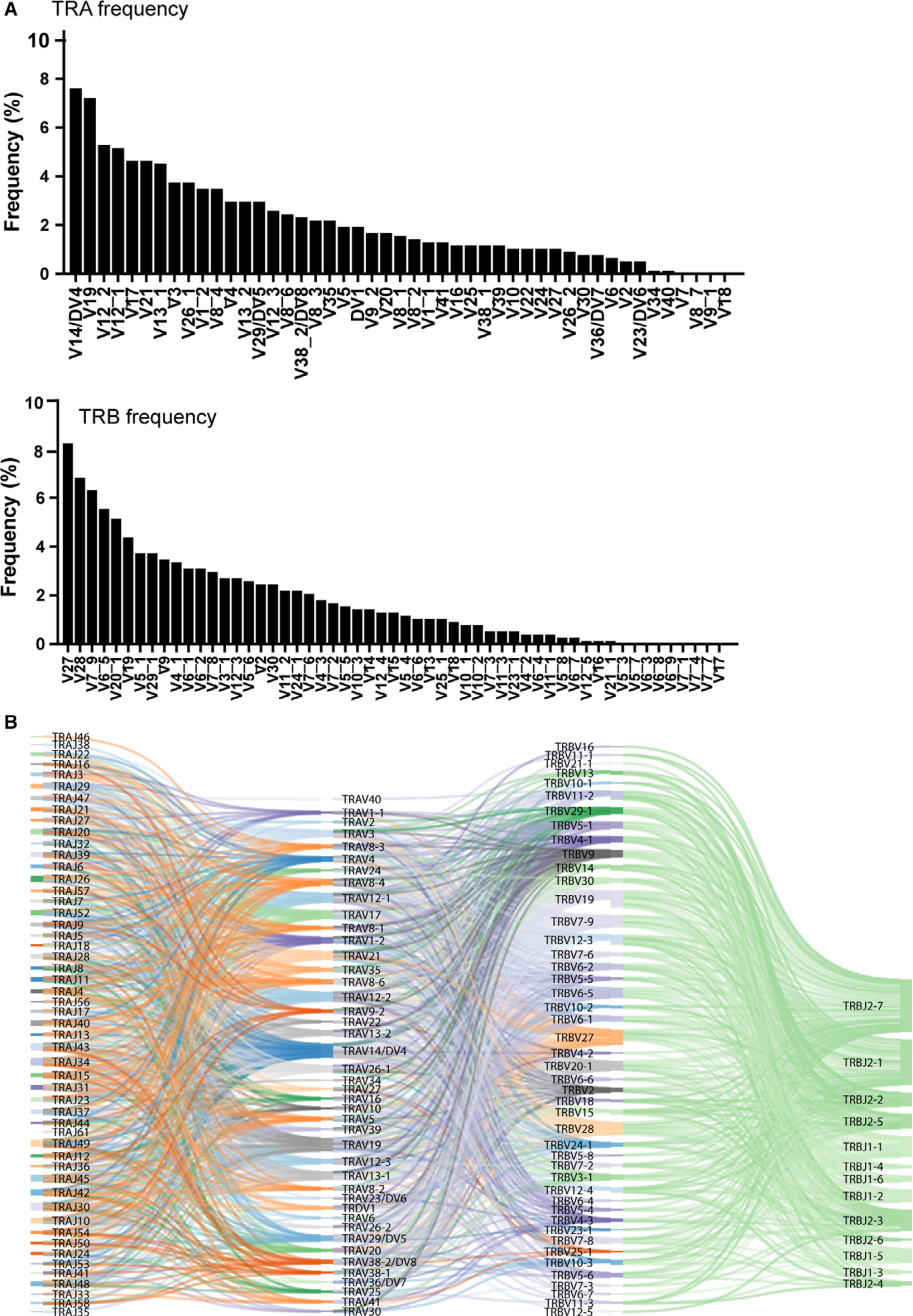
The utilization of TRAV and TRBV gene segment combinations for 776 unique sequences in human CD8 T cells. (A) The frequencies of TRA and TRB were calculated. (B) The usage of paired samples was plotted by the SankeyMATIC webtool (http://sankeymatic.com/build/). The color is the segment assignment the software displays for each gene, and the width of the bands is the frequency of the TRBV and TRAV genes connected by the bands co‐occurring in paired samples. The nomenclature for each gene is from the IMGT database.

**Figure 2:**
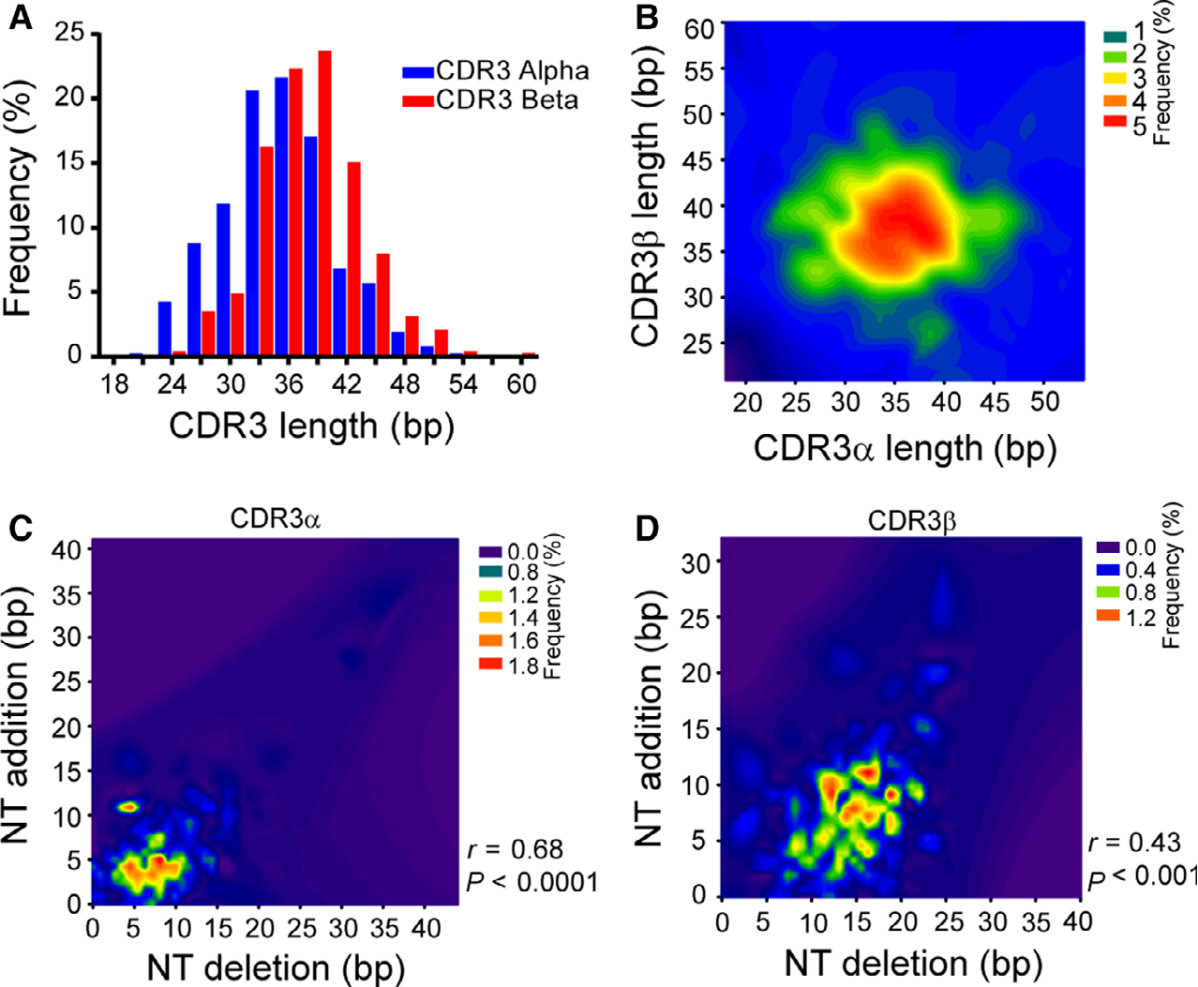
The CDR3 length distribution in paired samples. (A) The distribution of CDR3 length (bp) in both alpha (blue bar) and beta (red bar) chains. (B) The distribution of CDR3 lengths among the unique paired samples. The color indicates the frequency of each length. The correlation between nucleotide deletions and insertions in both alpha (C) and beta (D) chains. The color indicates the frequency, and the correlations were tested with the Spearman rank test.

**Figure 3:**
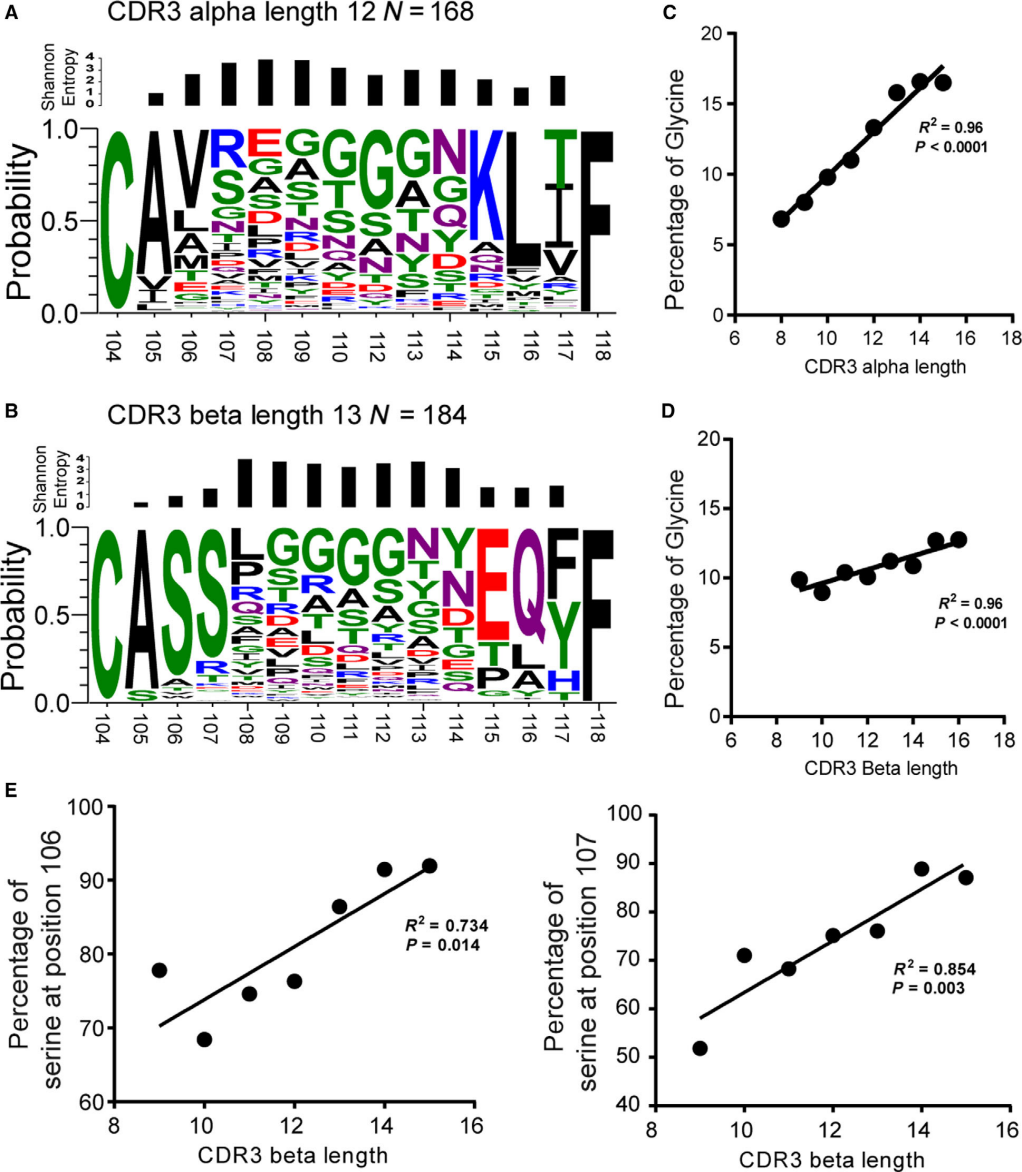
Amino acid residue distribution in paired CDR3 regions. The representative data for amino acid composition were displayed with weblogo 3.4 software for both the alpha (A) and beta (B) chains. The size of the letters represents the frequency of the amino acid at each position. (C) and (D) show the frequency of Glycine usage and the correlation with CDR3 length for both alpha and beta CDR3, respectively. (E) shows the frequency of serine usage in the CDR3 base.

Figure 1B was plotted by the SankeyMATIC webtool (http://sankeymatic.com/build/). The color represented different genes, and the width of the line indicated the frequency of the pairing between alpha and beta chains. Figure 2B–D was plotted by SigmaPlot heatmap function (Systat, San Jose, CA, USA), and the color indicated the frequency. Figure 3A,B was generated by WebLogo (https://weblogo.berkeley.edu/logo.cgi).

### Statistical analyses

Differences between groups were considered to be significant at a *P* value of < 0.05. Statistical analyses were performed with graphpad prism 7.0 (GraphPad Software, Inc., San Diego, CA, USA). The random sum of CDR3 alpha and beta lengths was generated with 602 176 different values, simply, in random setting, one value from alpha chain sum with 776 possible combinations from beta chain and vice versa, so we can get 602 176 values for random sum of CDR3. All the values and also the distribution of CDR3 length are shown in Table S5.

## Results

### A dataset of paired CDR3αβ segments in human CD8+ T cells

We obtained 776 unique pairs of sequences that represent functionally paired CDR3α and CDR3β segments expressed in human CD8+ T cells from a public dataset 15. All relevant patient and sample dataset information was clarified in a previous study and through personal contact 12. On average, each of the four unrelated donors with a similar age yielded 194 (±34.8) unique paired sequences from CD8+ T cells. Altogether, the sequence pairs covered 35.1% of possible functional gene combinations (total 46 TRAV and 48 TRBV functional genes from the IMGT database). Among all Vα segments, V7, V8‐7, V9‐1, V18, V34, and V40 were not detected in our sample (Fig. 1A), and among the Vβ segments, V5‐3, V5‐7, V6‐3, V6‐8, V6‐9, V7‐1, V7‐4, V7‐7, and V17 (Fig. 1A) were not detected, which is consistent with results from previous studies 17, 18, 19. We next analyzed the distribution of TRAV–TRBV pairing in our dataset, and we found the frequency of each specific TRAV–TRBV combinational usage varied between 0.18 and 2.37%, as shown in Fig. 1B. Interestingly, we found there is dominant usage of TRAV or TRBV genes in individual alpha or beta chain shown in Fig. 1A, and TRBJ2‐7 and TRBJ2‐1 genes were more frequently used compared with other TRBJ genes. Our dataset represent 35.1% of all TRAVs/TRBVs possible function gene combinations. Interestingly, when we anayzed the usage frequency of the combination of TRAVs/TRAJs with the paired TRBVs/TRBJs, the results indicated that the usage was evenly distributed in paired samples (Fig. 1B). Of note, we found that a novel recombination of TRDV1 segments paired with different Vβ segments, which account for 2.1% in our current dataset, although the functions of those combinations still remain unclear.

### Relative constrained length distribution of paired CDR3 in TCR α and β chains

Previous analyses have shown that the lengths of human CDR3s were normally distributed 20, 21. Consistent with those studies, we also found in our study that the CDR3 length followed a Gaussian distribution in both chains (α: *R* ^2^ = 0.97, β: *R* ^2^ = 0.99, nonlinear regression). The CDR3α length was distributed with a mean of 35.1 ± 5.8 nucleotides (range 18–54), whereas the CDR3β length was significantly longer (*P* < 0.001, Mann–Whitney test) with 38.0 ± 5.4 nucleotides (range 21–60), as shown in Fig. 2A. However, the distribution of CDR3α/β length remains unclear in the paired samples. A previous study using a small number of samples and a mathematic method predicted that the sum of CDR3 alpha and beta lengths has a relatively narrow distribution, and two mechanisms have been proposed, including one in which there was a long α chain with short β chains in CDR3 and vice versa. The other mechanism indicated that individual α and β CDR3 lengths have an even, narrow length distribution 22.

We hypothesize that if the first mechanism was applicable, we should be able to see a negative correlation or at least a trend between the lengths of CDR3α and CDR3β chains, and the Spearman correlation test showed that there was no significant correlation, or even a trend, between the CDR3 lengths of the alpha and beta chains (*R* = 0.01, *P* = 0.76). Indeed, the sum of CDR3 lengths also fit the normal distribution very well (Fig. S4), and moreover, the high frequency of the CDR3 length displayed a peak between 10 and 14 amino acids for both chains shown in Fig. 2A,B (27 ± 3aa). The skewness and kurtosis were the parameters for measuring normal distribution. Our analysis suggested that the distribution of the combined length and alpha chain was more sharply peaked and less skewed than the beta chain (Table 1), implying that beta chains have more diversity in terms of CDR3 length, and again confirms that CDR3 length in alpha and beta chains is normally distributed. We also calculated the theoretical length distribution of combined alpha and beta chains based on a random assortment (Table S5), and when we superimposed two datasets, we found the experimental CDR3 length had a very similar distribution to the predicted length (Fig. S4). We next examined which factors might contribute to shaping the CDR3 length distribution. One of the factors that created CDR3 diversity is from junctional region, which was generated by nucleotide deletions and insertions (indels). Here, we also hypothesized that indels could shape the CDR3 length distribution. The lengths of indels in CDR3α sequences ranged from 0 to 15 nucleotides, with a small fraction having a length of 20 or greater. In contrast, the indel lengths of CDR3β ranged from 5 to 20 nucleotides and thus were spread less widely compared to CDR3α (Fig. 2C,D). Next, we assessed the correlation between indels and CDR3 length for both chains. The Spearman rank testing showed that indels were significantly and positively correlated with length in both CDR3α (*r* = 0.68, *P* < 0.0001) and CDR3β (*r* = 0.43, *P* < 0.001), which suggests that nucleotide deletion and insertion could shape the CDR3 length distribution. TRBD genes are one of the important factors to contribute to the diversity of CDR3β, so we then assessed whether TRBD genes also contribute to the length of the CDR3 region. We found that the length of the TRBD region was significantly correlated with the length of the CDR3 region (Fig. S1A).

**Table 1:** Means, standard deviations, and descriptive parameters defining the Gaussian‐like distribution for human alpha and beta chain CDR3 length distributions

	Mean	SD	Variance	Skewness	Kurtosis
CDR3 alpha	12.71	1.93	3.72	0.20	0.05
CDR3 beta	14.66	1.78	3.18	0.28	0.58
CDR3 sum	27.37	2.63	6.92	0.14	0.08
CDR3 sum predicted	26.37	2.63	6.91	0.17	0.13

### Nonrandom amino acid distribution in the CDR3αβ region

To further investigate the diversity and distribution of amino acids in the CDR3 regions, we generated amino acid distributions using the WebLogo application (http://weblogo.berkeley.edu/logo.cgi) and expressed the variability at the given positions of each CDR3α and CDR3β sequence as Shannon entropy, which are shown in Fig. 3A,B. All the CDR3 amino acid sequences are listed in Table S2 and Table S3 for alpha and beta chains, respectively. The examination of Shannon entropies revealed that CDR3α positions 105–107 showed greater diversity than positions 115–117. In the CDR3β base region, the diverse positions were more or less evenly distributed. Amino acid residues at CDR3β positions 105–107 (ASS) and 115–117 (EQY/F) were conserved. A similar level of conservation was seen at CDR3α positions 115–117 (KLI/T), whereas positions 105‐107 (AV/LX) were mostly occupied by hydrophobic amino acids (Figs 3A,B, S2–S3). In contrast, CDR3β contained polar serine residues at these positions. Interestingly, we observed that the frequency of glycine increased in a position‐ and length‐dependent manner in both the CDR3α and CDR3β regions (Fig. 3C,D). In addition, the frequency of serine at positions 106 and 107 was positively correlated with CDR3β length (Fig. 3E).

### Physicochemical characteristics of paired CDR3αβ regions

**Figure 4:**
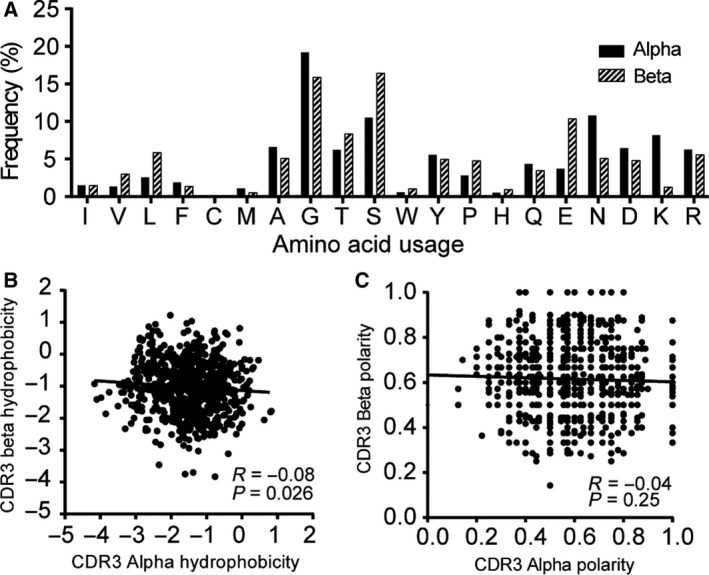
Paired CDR3α and CDR3β sequences in human CD8 T cells. The frequency of amino acid usage was calculated for positions 107–115 for alpha (black bar) and beta (white bar) chains, and the χ^2^ test was used to calculate the significance. (B) and (C) show a comparison of hydrophobicity and polarity in both alpha (white) and beta (blocked) chains. Hydrophobicity (B) and polarity (C) are defined in the text, and each symbol represents one CDR3αβ pair (*N* = 776). The correlation was assessed by using Spearman's rank correlation coefficient.

Previous studies have shown that the first and last three residues of CDR3 are buried and are not directly engaged in antigen binding 16, 23. We thus focused on the amino acid residue composition at positions 107–115, which were located in the surface‐exposed loop of the CDR3 regions (Table S4). The amino acid composition at these positions differed significantly between the CDR3α and CDR3β regions (*P* < 0.0001, χ^2^ test, 19 degrees of freedom; Fig. 4A). We further analyzed the genetic code distribution of each amino acid, and the results showed that there are very different trends of codes between alpha and beta chains; for example, glycine was mainly coded by GGA in alpha chain, while it was coded by GGG in beta chain (Fig. S1B). Next, to gain further insight into possible intrinsic pairing rules, we analyzed paired samples by the IMGT amino acid properties for hydrophobicity and polarity. We found no significant correlations, except for the hydrophobicity of CDR3α and CDR3β regions, which were weakly, but significantly, correlated (Spearman r = −0.08, P = 0.026; Fig. 4B,C).

## Discussion

The interface between a αβ TCR and peptide–MHC represents the structural solution to almost 450 million years of coevolution 24. The peptide specificity of the TCR is primarily determined by CDR3 loops of the α/β chains. Unlike other related work 19, 25, we comprehensively analyzed multiple aspects of CDR3 regions, including CDR3 length distribution, nucleotide deletion and insertion, and amino acid usage in the paired TCR αβ chains of single cells.

We found that certain TRBV genes, such as TRBV27, TRBV28, TRBV7‐9 and TRAV13‐1, and certain TRAV genes (TRAV19 and TRAV13‐2) are common while others are quite rare (Fig. 1A) and in addition, the usage of TRBJ 2‐7 and TRBJ2‐1 is not random (Fig. 1C). These observations were consistent with previous large data sets which only analyzed either alpha or beta chains, suggesting that usage and distribution of TRBV and TRAV in our dataset is consistent with other sets 17, 19, 26, 27. The reasons for bias are not clearly understood but are likely due to a combination of proximity effects and recombination signal sequence compatibilities which can influence TCR development and selection 28.

Previous studies have shown that the length of the CDR3 region follows a normal distribution; however, little is known about paired samples 18. Johnson and Wu were the first to observe unpaired TCR α and β chain datasets in a small mouse and humans. They attributed their results to the random association of individual TCR CDR3 α and β chains that were narrowly distributed rather than a biological selective matching mechanism including a long TCR CDR α(β) chain with short TCR CDR β(α) chains 25, 29. This was supported by recent computational modeling analysis in a human T‐cell receptor repertoire study 30. To the best of our knowledge, this is the first time that their rarely cited findings have been confirmed and experimentally supported at the single‐cell level. Considering that only 21–34% of the αβ TCR surface interacts with a peptide–MHC complex and that thymic selection does not appear to play a role in CDR3 length distribution of CD8+ T cells 1, it is possible that the constrained CDR3 length distribution might result from the restriction of peptide–MHC interaction during competitive antigen‐driven coevolution. Indirect support for this interpretation comes from different CDR3 lengths and highly variable H and β chains of length γδ TCRs, which recognize antigens comparable to immunoglobulins.

Glycine is known to contribute to the flexibility of the CDR3 loops in both TCRs and BCRs. In line with previous reports, we found that the frequency of glycine use was higher in TCR β than in TCR α chains 31. In addition, our analysis showed that a length‐ and position‐dependent increase in glycine usage in both TCR α and β chains may confer cross‐reactivity and the ability to recognize mutant or different pathogens 15. In addition, TCR polyspecificity is an intrinsic property of TCR recognition, which has been defined as the ability to recognize multiple distinct peptide/MHC ligands 32, 33. The amino acid distribution in the CDR3 region must play a critical role. A study has shown that the antibody has higher frequency of alanine usage in their CDR3 region which contributed more flexibility of BCRs 34. The molecular basis for TCR polyspecificity is currently not well defined. Studies showed that a given TCR can adopt large conformational changes of one CDR3 loop to productively interact with different pMHC complexes 4. Aside from glycine, the WebLogo analysis revealed the conserved small amino acid motif CASS at the base of TCR CDR3β regions. Considering that TCR mobility is dependent on the intrinsic flexibility of CDR3α and CDR3β regions, the frequent use of small and flexibility‐mediating amino acids at the CDR3β base may explain in part why CDR3β chains move on a faster timescale than CDR3α chains, which contain a mostly hydrophobic A[VL]X motif at the base region 31, 35. The CDR3α and CDR3β regions differ in their Shannon entropies as well. CDR3α positions 105–107 showed greater diversity than positions 115–117, whereas the CDR3β base region showed little diversity. Since positions 105–107 of CDR3α chains are encoded by TRAV gene segments and TRAJ for positions 115‐117, CDR3α diversity appears to depend more on the usage of TRAV rather than TRAJ. A similar TRAV‐driven combinational diversity was also observed in mice 36.

The intrinsic pairing properties of CDR3αβ chains include amino acid composition, hydrophobicity, and polarity. A study of CDR3 chains in CMV‐specific CTLs showed that the hydrophobicity of the TCR CDR3α and CDR3β chains was strongly and negatively correlated 16. This outcome contradicts our results, which showed only a very weak inverse association for hydrophobicity and polarity. The opposing findings represent biases in CDR3 chains expressed in CMV‐specific CTLs versus in naïve CD8+ T cells.

Interestingly, we observed that 2.1% of TCR TRDV1 segments were paired with different TCR TRBV segments in this dataset; however, we do not know the specificity of those TCRs. Recently, several studies in HIV‐1 have found HIV‐1‐specific CD8 T cells expressed these recombination 37, 38, and the crystal structures of TCR‐pMHC revealed that TRDV1/TRBV TCR was similar to the TRAV/TRBV structures 39.

In summary, we presented a comprehensive picture of the length distribution, amino acid use, and properties of CDR3 regions from paired CD8 T cells. We found that the paired CDR3 lengths were narrowly distributed despite their diversity. Second, the usage of amino acids in the CDR3 loop was not random and was significantly different between TCR α and β. Our study provided the basic features of the CDR3α/β region, which provided basic knowledge of T‐cell immunology and potential applications for TCR‐based immunotherapy. We plan to sequence TCR repertoire to further study the features of the CDR3 region in antigen‐specific T cells in cancer study.

However, the limitation of our current study is that we included only a limited number of paired TCR samples from one study in our analysis. Since emulsion PCR methods were created, single‐cell TCR/BCR sequencing combined with next‐generation DNA sequencing has been developed and applied to the paired analysis of TCR repertoire 40, 41, 42. We expect that our initial dataset will become the seed for a larger paired TCR αβ chain reference dataset generated from single cells. This dataset may help solve the remaining uncertainties in TCR pairing dynamics and properties and provide a base for TCR modifications applied in adoptive immunotherapy.

## Conflict of interest

The authors declare no conflict of interest.

## Author contributions

KY and JS conceived and designed the study, analyzed the data, wrote the paper, prepared figures, and reviewed drafts of the paper. QY conceived and designed the study, discussed the data, wrote the paper, and reviewed drafts of the paper. DL wrote the paper and reviewed drafts of the paper.

## Supporting information


**Fig. S1**. CDR3 length and amino acid genetic codes. (A) The correlation between TRBD nucleotide and CDR3 length, the spearman correlation was tested. (B) The gene code for each amino acid was listed and color just to distinguish different codes within amino acid.

**Fig. S2.** Amino acid residue distribution in paired CDR3 alpha regions. The representative data for amino acid composition were displayed by WebLogo 3.4 software in alpha chains. The size of the letter represents the frequency of the amino acid at each position.

**Fig. S3.** Amino acid residue distribution in paired CDR3 beta regions. The representative data of amino acid composition were displayed by WebLogo 3.4 software in beta (B) chains. The size of the letter represents the frequency of the amino acid at each position.

**Fig. S4.** Superimposition of CDR3 length distribution from the sum results from experimental and predicted results.

**Table S1.** Sample sequence in this study analyzed by IMGT (n = 776). **Table S2.** Amino acids of the CDR3 alpha chain (n = 776). **Table S3.** Amino acids of the CDR3 beta chain (n = 776). **Table S4.** Biochemical properties of amino acids for both the alpha and beta chains (n = 776). **Table S5.** The sum of CDR3 length in alpha and beta chains from experimental and predicted results.
