# Supplementary figures and images for: Comparative analysis of CDR3 regions in paired human αβ CD8 T cells
Source: FEBS Open Bio. 2019 Jul 12;9(8):1450–9. doi: 10.1002/2211-5463.12690 (PMC6668380; doi:10.1002/2211-5463.12690)

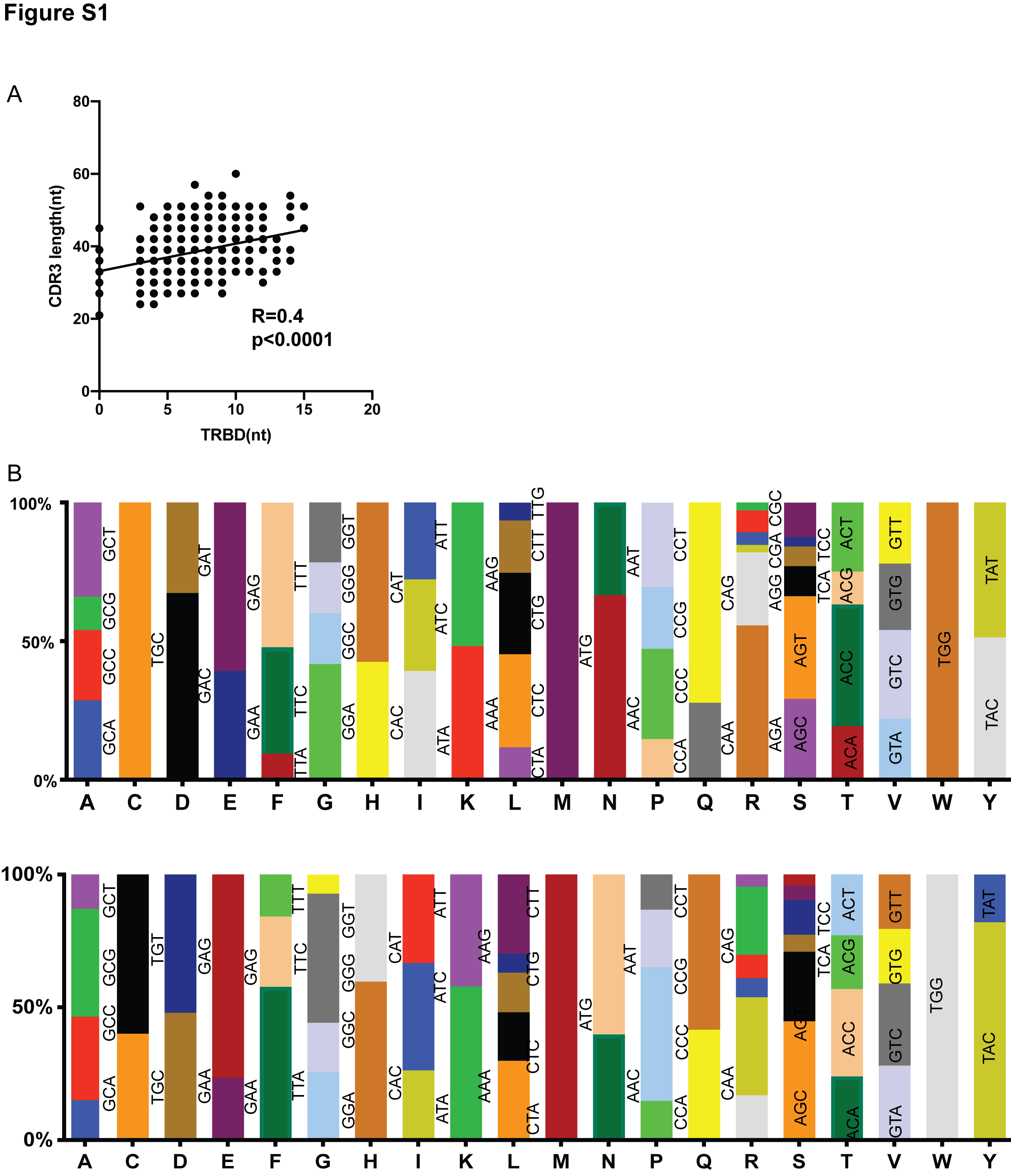

Supplement: Supplementary file 1 — Fig. S1. CDR3 length and amino acid genetic codes. (A) The correlation between TRBD nucleotide and CDR3 length, the spearman correlation was tested. (B) The gene code for each amino acid was listed and color just to distinguish different codes within amino acid. [file FEB4-9-1450-s001.tif]

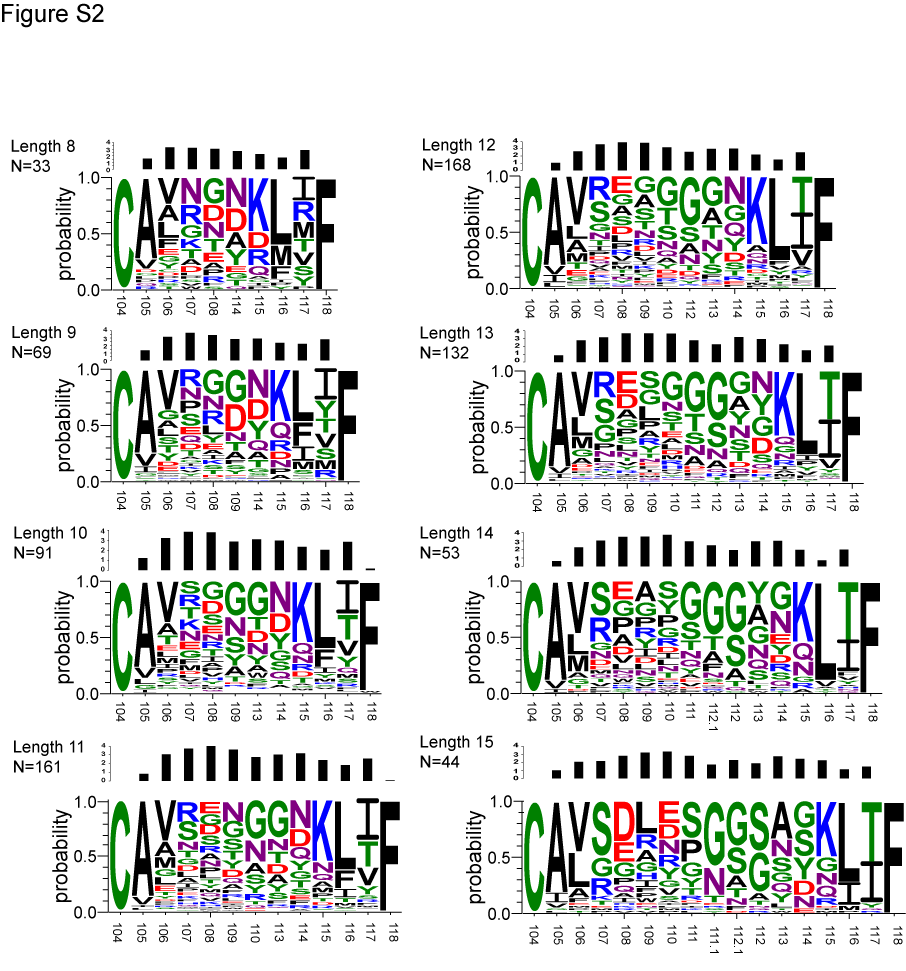

Supplement: Supplementary file 2 — Fig. S2. Amino acid residue distribution in paired CDR3 alpha regions. The representative data for amino acid composition were displayed by WebLogo 3.4 software in alpha chains. The size of the letter represents the frequency of the amino acid at each position. [file FEB4-9-1450-s002.tif]

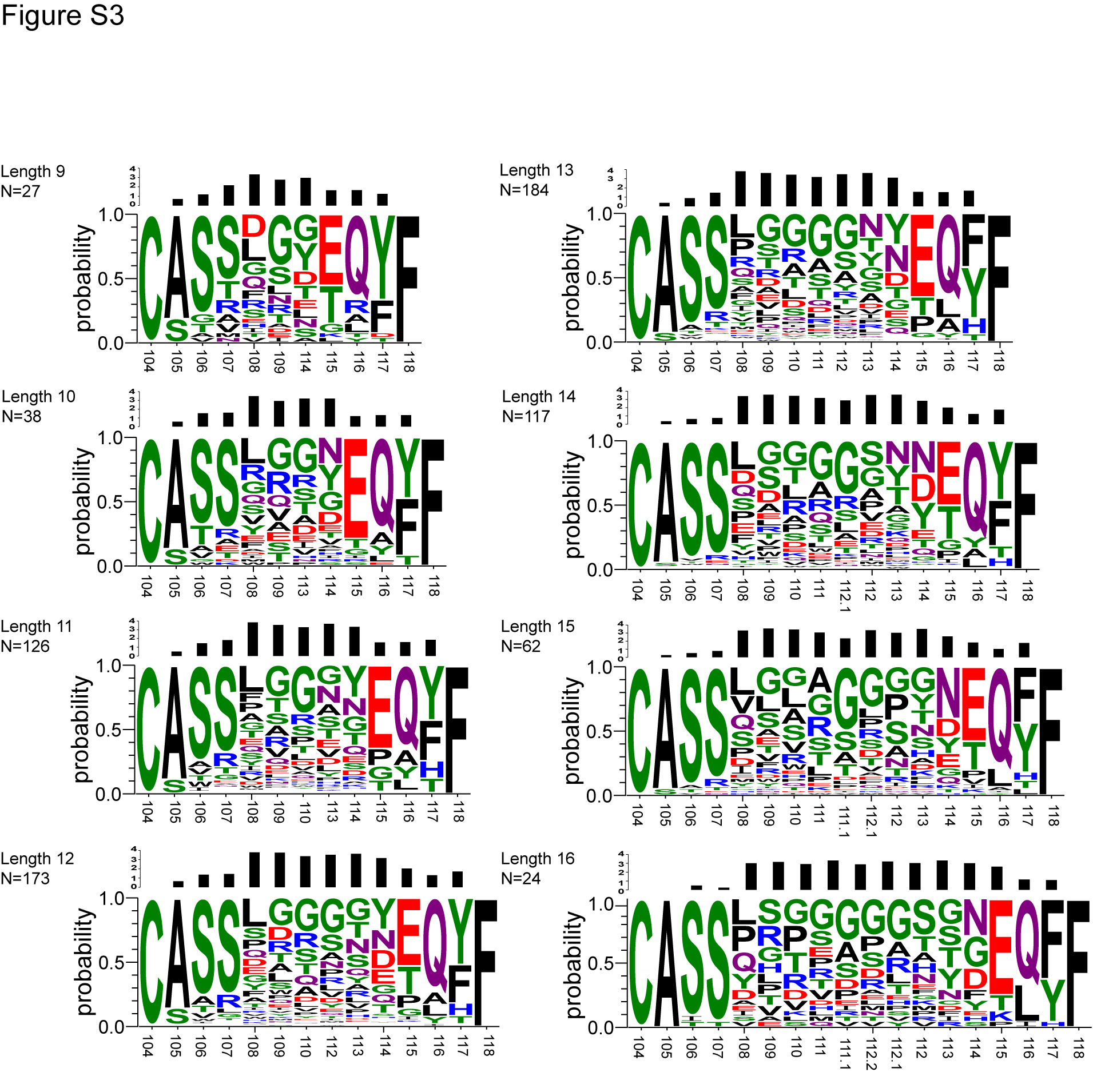

Supplement: Supplementary file 3 — Fig. S3. Amino acid residue distribution in paired CDR3 beta regions. The representative data of amino acid composition were displayed by WebLogo 3.4 software in beta (B) chains. The size of the letter represents the frequency of the amino acid at each position. [file FEB4-9-1450-s003.tif]

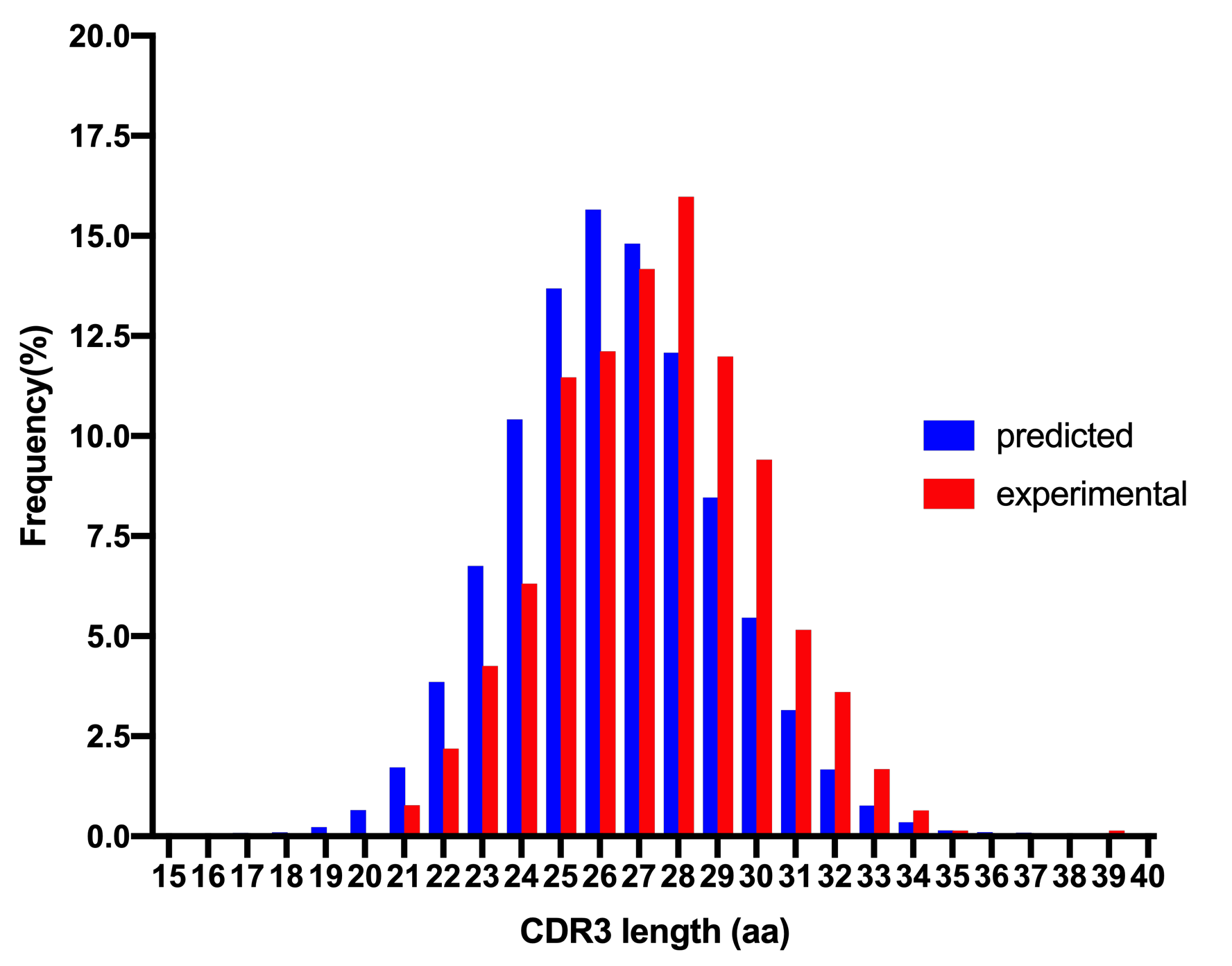

Supplement: Supplementary file 4 — Fig. S4. Superimposition of CDR3 length distribution from the sum results from experimental and predicted results. [file FEB4-9-1450-s004.tiff]
